# Human Papillomavirus and Tonsillar and Base of Tongue Cancer

**DOI:** 10.3390/v7031332

**Published:** 2015-03-20

**Authors:** Torbjörn Ramqvist, Nathalie Grün, Tina Dalianis

**Affiliations:** Department of Oncology-Pathology, Karolinska Institutet, Cancer Center Karolinska R8:01, Karolinska University Hospital, 171 76 Stockholm, Sweden; E-Mails: Torbjorn.Ramqvist@ki.se (T.R.); Nathalie.Grun@ki.se (N.G.)

**Keywords:** human papillomavirus, tonsillar squamous cell carcinoma, base of tongue squamous cell carcinoma cancer, clinical outcome, oral HPV prevalence

## Abstract

In 2007, human papillomavirus (HPV) type 16 was recognized as a risk factor by the International Agency for Research on Cancer, for oropharyngeal squamous cell carcinoma (OSCC), where tonsillar and base of tongue cancer (TSCC and BOTSCC) dominate. Furthermore, patients with HPV-positive TSCC and BOTSCC, had a much better clinical outcome than those with corresponding HPV-negative cancer and other head and neck cancer. More specifically, survival was around 80% for HPV-positive TSCC and BOTSCC *vs.* 40% five-year disease free survival, for the corresponding HPV-negative tumors with conventional radiotherapy and surgery, while this could not be observed for HPV-positive OSCC at other sites. In addition, the past 20–40 years in many Western Countries, the incidence of HPV-positive TSCC and BOTSCC has risen, and >70% are men. This has resulted in a relative increase of patients with HPV-positive TSCC and BOTSCC that may not need the intensified chemo-radiotherapy (with many more severe debilitating side effects) often given today to patients with head and neck cancer. However, before tapering therapy, one needs to enable selection of patients for such treatment, by identifying clinical and molecular markers that together with HPV-positive status will better predict patient prognosis and response to therapy. To conclude, there is a new increasing group of patients with HPV-positive TSCC and BOTSCC with good clinical outcome, where options for better-tailored therapy are needed. For prevention, it would be of benefit to vaccinate both girls and boys against HPV16 infection. For potential future screening the ways to do so need optimizing.

## 1. Introduction

Twenty years ago in 1995, the International Agency for Research against Cancer acknowledged an association between human papillomavirus (HPV) type 16 and cancer of the cervix, uteri, and other anogenital cancer [1]. It took another 12 years for HPV16 to be recognized as a risk factor for oropharyngeal squamous cell carcinoma (OSCC) in 2007, dominated tonsillar and base of tongue cancer (TSCC and BOTSCC) and where HPV is mainly found [2,3,4,5,6,7]. The most notable difference between HPV-positive TSCC and BOTSCC compared to their HPV-negative counterparts and other head and neck squamous cell carcinoma (HNSCC) is the better clinical outcome (80% *vs.* 40% five-year disease specific survival) [2,3,4,6,7,8,9]. This difference in clinical outcome has not been observed between HPV-positive and HPV-negative OSCC at other sites other than TSCC and BOTSCC [6]. In the past decades, the incidence of especially TSCC and BOTSCC (and thereby also OSCC) has increased in many Western countries, mainly due to a rise of HPV-positive TSCC and BOTSCC cases and >70% are men [8,10,11,12,13,14,15,16,17,18,19,20,21]. HNSCC has poor prognosis in general and is now given more aggressive treatment with more intensified chemotherapy and radiotherapy, leading to more adverse side effects [21]. Such therapy may not be beneficial for most patients with HPV-positive TSCC and BOTSCC, where 80% of the patients survived, before treatment was intensified, and when given only conventional radiotherapy alone, with the addition of surgery if needed [2,3,4,6,7,8,9,19]. Here, differences between HPV-positive and HPV-negative TSCC and BOTSCC and issues of finding biomarkers in HPV-positive TSCC and BOTSCC useful for predicting which patients may have a good response to therapy and be eligible for de-escalated therapy trials are described. Data on oral HPV infection, effects of HPV vaccination, and potential screening for TSCC and BOTSCC are also discussed.

## 2. Human Papillomavirus (HPV) and Disease and Cancer

There are >170 HPV types, with the majority found in the skin (cutaneous HPV types), but many also found in mucous tissues (mucosal HPV types) and where clearly the vast majority cause only asymptomatic infections [22,23]. A phylogenetic tree based on the homologous nucleotide sequence of the major capsid protein L1 groups the different HPV types into five genera—alpha, beta, gamma, mu and nu [23]. The mucosal types are included in genera alpha and the others mainly consist of cutaneous types [23].

Mucosal HPV types can be divided into high-risk (HR) types that have a clear and well-recognized potential to cause cancer, or low-risk types (LR) that are very rarely observed in cancer [22,23]. The best-known association between HR-HPVs and cancer is that of HPV and cancer of the uterine cervix [22]. However, HPV is also associated with vulvar, vaginal, penile and anal cancer, and since 2007, HPV16 has also been acknowledged to be a risk factor for OSCC, where tonsillar and base of tongue cancer dominate [5,22,23,24,25]. Furthermore, in addition to HPV16, HPV33, HPV35 and others (also found in cervical cancer) have been observed to contribute to OSCC [4,7,15,24,26]. LR-HPV types are not associated with cancer development in general, but are often found in benign genital lesions, such as condylomas and recurrent respiratory papillomas [22,23].

Cutaneous HPV types are best known to cause skin warts, but multiple skin cancers may emerge from verruca-like papillomatous lesions in Epidermodyplasia vercucciformis (EV) patients that are especially sensitive to infections with e.g., HPV5 and 8 [1,5,23]. Whether HPV is responsible for other squamous cell carcinoma of the skin is however still a question of debate.

HPVs have double stranded circular DNA genomes of around 7.9 kb. The genome, including a non-coding control region (NCCR), an early and a late coding region, is enclosed together with histones within a 52–55 nm virion [23,24]. The regulatory proteins E1-E2, E4-E7 important for gene regulation, replication and pathogenesis are coded by the early region, while the two structural proteins L1 and L2 responsible for the viral capsid are encoded by the late region [23,24]. E6 and E7 regarded as oncogenes in HR-HPV types and have high affinity to p53 and pRb, respectively, and are without doubt of relevance for immortalization and transformation [23,24]. The binding of E6 to p53 causes its degradation, preventing e.g., control of DNA damage and cell repair or apoptosis, while the binding of E7 to Rb and abrogates deregulation of cell cycle control [1,18,19,23,24]. The latter, also results in an increase in the expression of the cyclin dependent kinase inhibitor p16^INK4a^ [23,27]. Overexpression of p16^INK4a^ was in the past used as a surrogate marker for presence of HPV in OSCC [24,27]. Today, neither overexpression of p16^INK4a^ or the presence of HPV DNA alone, are regarded as sufficient to point that a tumor is caused by HPV [24,28]. However, presence of HPV DNA combined with p16^INK4a^ overexpression is almost as sensitive as using the golden standard, *i.e.* analyzing for presence of HPV E6 and E7 mRNA [24,28]. The L1 major capsid protein contributes to around 80% of the viral capsid and can spontaneously self-assemble into virus-like particles (VLPs) under specific conditions [22,23,29]. Todays’ HPV vaccines Cevarix (GMK) and Gardasil (Merk) consist of VLPs from different HPV types and both contain HPV16 and 18 VLPs, while the latter also contains VLPs also of HPV6 and HPV11 [30,31]. These vaccines have been shown to be very efficient against cervical HPV infection, and also very likely efficient against oral HPV infection [30,31,32,33,34,35].

## 3. TSCC, BOTSCC and HPV and Its Influence on Clinical Outcome and Number of Cases

OSCC includes not only TSCC and BOTSCC, which accounting for 80% of the cases, but also cancer of the walls of the pharynx and the soft palate [36]. However, it becomes evident that the presence of HPV is highest in TSCC and BOTSCC (79% and 73%, respectively), which both contain lymphoid tissue and together contribute to Waldeyers ring, while in other OSCC the presence of HPV is lower (17%) [6,8,17]. This is not always appreciated, and it is likely that this distinction must also be made more clearly before selecting patients for clinical studies. Furthermore, HPV16 is present in >90% of the HPV-positive TSCC and BOTSCC cases, and even more common than in cervical cancer, where HPV16 accounts for 50%–55% of the cases; moreover, most of the patients are men [1,2,3,4,5,6,7,23,24,26]. Taken together, this is also of importance when reflecting over HPV vaccination against these tumors.

The most prominent difference between HPV-positive and HPV-negative TSCC and BOTSCC is that patients with HPV-positive tumors have a much better clinical outcome than those with HPV-negative tumors and other head and neck cancer, including OSCC other than TSCC and BOTSCC (80% *vs.* 40% five-year survival) [3,4,7,8,9]. In the literature, similar findings with regard to clinical outcome are found for HPV-positive and HPV-negative OSCC, which is not surprising, since the numbers of OSCC cases outside the tonsil and base of tongue at other subsites are limited and do not change the general trend [4,6,36].

Patients with HPV-positive TSCC and BOTSCC as compared to those with HPV-negative tumors are also often somewhat younger, more often non-smokers, have smaller tumors, but have a higher tumor stage, since many patients also have nodal disease [2,3,4,6,7,8,9,37]. Nonetheless, the latter still does not necessarily affect the better survival among patients in the HPV-positive group [2,3,4,6,7,8,9,37].

Most HPV-positive TSCC and BOTSCC independent of episomal/and or integrated HPV genomes, exhibit E6 and E7 mRNA expression; with p53 expression more often, being normal and with 16^Ink4a^ overexpressed in most cases, in contrast to that observed in HPV-negative TSCC and OSCC [23,37,38,39,40]. HPV-positive TSCC and BOTSCC is also generally less differentiated; more frequently aneuploid compared to HPV-negative OSCC; and chromosome 3q often amplified similar to cervical cancer, but amplification of chromosome 3q did not further influence clinical outcome [41,42]. HPV has repeatedly been a favorable prognostic marker, independent of tumor stage, age, gender, differentiation, or DNA ploidy, and in never-smokers clinical outcome has generally been found to be even better [2,3,4,6,7,8,9,37,41,42].

Today the golden standard of HPV-positive status in TSCC/BOTSCC/OSCC is the presence of E6 and E7 mRNA expression by RT-PCR, suggested to be associated with functional HPV expression [28]. Notably, the combined presence of HPV DNA tested by PCR and p16 overexpression is now more and more widely recognized as very close to the golden standard in TSCC and BOTSCC and especially useful for analysis in formalin fixed paraffin embedded (FFPE) tumor samples [28]. Nonetheless, in the past many ways of defining HPV-positive status have been used, including different types of PCR assays and primers, which may have given some variations in the results obtained. [4,7,27,28,37,43,44,45,46,47,48]. Moreover, HPV prevalence varies not only due to the methodology used, but also depending on geographical location and time period of investigation. Numerous studies have indicated an increase in HPV prevalence in TSCC, BOTSCC and OSCC the past decades and this has certainly contributed to the increased incidence of these tumors that has been reported from many Western countries [8,10,11,12,13,14,15,16,17,18,19,20,21].

For example, between 1970 and 2002, a three-fold increase in both the incidence and prevalence of HPV (both in women and men) was observed in TSCC in Stockholm, Sweden and this lead to the hypothesis that HPV was responsible for the increase in incidence of TSCC [15]. This was followed by additional reports again from Stockholm, showing that HPV-positive TSCC had increased with a seven-fold doubling per decade 1970–2007, while HPV-negative cancer had decreased and analogous changes were also reported for BOTSCC [8,14,17]. The most recent report from Sweden, shows a continued increase in incidence of TSCC and BOTSCC, however, while the number of HPV-positive TSCC cases remains high in the Stockholm area, it has not increased the past five years [49]. Whether this trend is temporary or not needs to be investigated further.

Parallel to the Stockholm studies, an emerging epidemic of HPV associated OSCC was suggested in the US, and in 2011 in the U.S. a rise in the incidence of HPV-positive OSCC and a decline in HPV-negative OPCC was also reported for the past decades [11,12,16,21]. Furthermore, during the same period accumulating reports from many Western countries, such as Scotland, the UK, and the Netherlands, conveyed an increasing incidence of OSCC, while in Eastern Denmark an increase in HPV-positive TSCC was described [10,13,20,50].

As already mentioned, the increase in incidence of TSCC, BOTSCC and OSCC has been suggested due to a rise in HPV-positive cases and changes in life style, and there is a significant correlation between HPV-positive OSCC, early sex debut, and numbers of oral or vaginal partners [51]. However, it has also been shown that oral-to-oral contact and HPV-transmission at birth may also result in oral HPV infection [52,53]. Clearly, in many Western countries, the numbers of HPV-positive TSCC and BOTSCC, where most patients are men, are still increasing, although new trends may be appearing [8,10,11,12,13,14,15,16,17,18,19,20,21,49,50].

## 4. TSCC, BOTSCC, HPV and Other Biomarkers and Treatment

HPV-positive TSCC and BOTSCC contribute to an increasing proportion of HNSCC in recent years [19]. Furthermore, this group of patients with a favorable clinical outcome already with conventional radiotherapy, may in most cases not need the intensified chemo-radiotherapy, with more side effects and increasing expenses for society that is given to head- and neck-cancer today [19,24]. Thus, it is imperative to distinguish patients that need intensive therapy from those who do not. For this purpose, it is important to combine positive HPV-status with additional biomarkers in order to better predict response to therapy and only select patients with a very probable good response rate to randomized trials with lessened therapy.

It has been shown that for TSCC and BOTSCC, the presence of HPV DNA/RNA and p16 overexpression are very good prognostic markers especially when combined with accurate data on that the patient being a never smoker [9,28,37]. In fact even the quantity of smoking, *i.e.*, package years, was also important [37]. However, none of these factors distinguish 100% of the patients and additional markers are therefore needed.

Additional biomarkers have been investigated. It has been reported that absent/low expression of MHC class I, CD44, CD98, LMP7, or LMP10 intensity staining, or absence of HLA-A*02, or high LRIG1 expression improved prediction of clinical outcome for patients with HPV-positive TSCC and BOTSCC [54,55,56,57,58,59,60,61,62]. Absence of HLA class I immunohistochemistry (IHC) staining, e.g., indicated a 95%–100% probability of a three-year disease free survival, but identified only around 20% of the patients with a good clinical outcome [56,57]. In addition, the high CD8+ tumor infiltrating lymphocyte (TIL) counts were also very favorable for patients with HPV-positive TSCC and BOTSCC as compared to the corresponding tumors with low CD8+ TIL counts, but was less sensitive [63,64].

That having high CD8+ TIL counts was favorable was expected, since an efficient immune response may result in a favorable clinical outcome of HPV-positive TSCC and BOTSCC [63,64]. More enigmatic was that HPV-positive TSCC and BOTSCC with absent HLA class I expression had good prognosis since HLA class I down-regulation abrogates the immune response, especially in absence of NK-cells [56,57]. Here, it is possible that HPV E5 and E7 expression contributes to HLA class I downregulation and that treatment increases HLA class I expression this way enhancing the immune response against these tumors [56,57].

Other biomarkers that have been investigated are miRNAs and data are accumulating, but stringent concordance between different studies has not been shown so far [65,66,67].

Clearly, additional molecular knowledge and ways to combine different markers, both clinical and molecular for prediction of clinical outcome in patients with HPV-positive TSCC and BOTSCC would be of great value.

## 5. Prevention of HPV-Positive TSCC and BOTSCC

HPV16 is the most common HPV type in the oral cavity in non-vaccinated individuals [68,69,70,71]. Oral HPV prevalence, including all HPV types, varies and has been reported to be 3%–9% in studies including individuals at all ages and when limited to unvaccinated youth [68,69,70,71]. Furthermore, it has been shown that women with a cervical HPV infection more often have an oral HPV infection [68].

In HPV vaccinated groups, oral HPV prevalence, especially HPV16, seems to be lower than in non-vaccinated groups suggesting a vaccination effect [32,33,34,35]. Thus vaccinating both girls and boys against HPV16 may be a good option not only to prevent cervical cancer but also to prevent the majority of HPV16 positive TSCC and BOTSCC in the future, especially since >70% of the latter comprise men.

For non-vaccinated individuals, other approaches may be of interest and screening may not be optimal for several reasons especially since the incidence of HPV-positive TSCC and BOTSCC is still relatively low [12,14,49]. Furthermore, testing HPV-prevalence in the oral cavity could result in an underestimation, e.g., due to saliva production, and some individuals may be reported as falsely negative because the obtained HPV signals are generally lower compared to those obtained for the cervical site [68].

Nevertheless, in the proportion of patients with HPV-positive TSCC and BOTSCC, it has been shown that the viral load of HPV16 in mouthwashes is considerably higher than that obtained in healthy youth and often comparable to that obtained in the cervix [72]. Whether a high viral HPV load is indicative of HPV-positive TSCC and BOTSCC needs to be investigated further. However, there is also the possibility to combine this approach with serology, where certain antibody profiles, especially the appearance of HPV16 E6 have been shown to predict risk for development of HPV-positive OSCC, and such antibodies are very seldom found in non-cancer patients [73,74]. Alternatively, serology could be used to identify patients at risk for OSCC and these patients could be followed using mouthwashes [72,73]. Using cytology has also been attempted, but not found very useful so far [67,75].

## 6. Conclusions

HPV-positive TSCC and BOTSCC have better clinical outcome than corresponding HPV-negative cancers and are increasing in incidence. Preventing spread of HPV16 infection by vaccination, and deescalating intensive therapy by using additional predictive markers to identify and select HPV-positive TSCC and BOTSCC patients eligible for randomized trials with lessened therapy are issues of importance.
